# The effects of sodium diethyldithiocarbamate in fibroblasts V79 cells in relation to cytotoxicity, antioxidative enzymes, glutathione, and apoptosis

**DOI:** 10.1007/s00204-012-0909-0

**Published:** 2012-08-08

**Authors:** I. Rahden-Staroń, E. Grosicka-Maciąg, D. Kurpios-Piec, H. Czeczot, T. Grzela, M. Szumiło

**Affiliations:** 1 Department of Biochemistry, Medical University of Warsaw, Banacha 1, 02-097 Warsaw, Poland; 2 Department of Histology and Embryology, Medical University of Warsaw, Chałubińskiego 5, 02-004 Warsaw, Poland

**Keywords:** GSH, TBARS, PC, Antioxidative enzymes, Apoptosis, V79 cells

## Abstract

Sodium diethyldithiocarbamate (DETC) is the main metabolite of disulfiram. Recently, we reported that mechanism of disulfiram cytotoxicity in V79 cells might be partially connected with thiol redox-state imbalance. Here, we examined the effect of DETC on the level of intracellular glutathione (GSH), protein oxidation (measured as PC—protein carbonyl content), lipid peroxidation (measured as TBARS—thiobarbituric acid reactive substances), antioxidant enzymatic defense, as well as on apoptosis. We used V79 Chinese hamster fibroblasts cells with and without modulated glutathione (GSH) level by *N*-acetyl-l-cysteine (NAC). We showed that treatment with DETC at concentrations that cause a moderate increase in thiol-state imbalance but not cell death stimulates oxidative stress measured as increased level of PC and TBARS, adaptive response of GSH-related enzymes and apoptosis. Our results show that cellular effects of DETC are partially attributable to the initial redox cellular state, since the increase of GSH level by NAC pre-treatment prevented the observed changes.

## Introduction

*N,N,*-Diethyldithiocarbamate (DETC) is a main metabolite of disulfiram (DSF) (Johansson 1992). DETC and its analogs are compounds with diverse applications, both as an important class of agricultural pesticides (dithiocarbamates) and as pharmacological agents. They are used against persistent *Mycobacterium tuberculosis* and as cancer chemo-preventive agent, potential adjunct to traditional oncological chemotherapy, along with protection against tissue toxicity of cisplatin treatment, and in experimental therapy of acquired immunodeficiency syndrome (AIDS) (Byrne et al. 2007; Pande and Ramos 2003; Pang et al. 2007). Although dithiocarbamates are known to display low acute and chronic toxicities in human and in experimental animals, the extreme reactivity mainly related to their metal-chelating ability (e.g. copper, iron, zinc) and high affinity for SH group-containing proteins underlies the wide range of their adverse effects (Orrenius et al. 1996; Somers et al. 2000). This chelating property of DETC is the basis for the therapy for metal intoxication in industrial conditions. DETC acts in cells as an inhibitor of superoxide dismutase (SOD) by chelating with intracellular Cu^+2^ (Lushchak et al. 2005). However, some biological actions of DETC may result from S-nitrosothiols elimination rather than SOD inactivation (Arnelle et al. 1997). DETC and its metabolite, carbon disulfide, are expected to provoke several side effects besides those related to the aversive reaction.

The free-radical chemistry of the dithiocarbamates appears to be more complex than that of other thiol compounds. Upon interaction with either superoxide (O_2_^−^), peroxyl (RO_2_), or hydroxyl (OH) radicals, DETC is oxidized to a thiyl radical, (Et)_2_NC(S)S, which can dimerize to form disulfiram (Kishore and Moorthy 1991; Mankhetkorn et al. 1994; Zanocco et al. 1989). Disulfiram regenerates DETC by oxidation of glutathione (GSH) to glutathione disulfide (GSSG) (Hosni et al. 1992). DETC was shown to possess a peroxidase-like activity, which utilizes exclusively glutathione as a substrate for the reduction of H_2_O_2_ and a limited number of organic hydroperoxides (Fitsanakis et al. 2002; Hosni et al. 1992). It is also the electron donor in the reaction catalyzed by glutathione peroxidase (GPx) (Fitsanakis et al. 2002) and is regenerated by glutathione reductase (GR) (Arthur 2000).

Since a mechanism of DETC action within a cell might be due to thiol redox-state imbalance, the main goal of the present study was to assess the effect of DETC on the level of intracellular glutathione, protein carbonyls, and lipid peroxidation levels, antioxidant enzymatic defense, as well as on apoptosis. We used the V79 cell line of Chinese hamster fibroblasts, well characterized and a common model system for cytotoxicity and mutagenicity studies (Bradley et al. 1981). Studies were performed in control V79 cells and in cells with modulated intracellular GSH level prior to drug exposure. Intracellular GSH was increased by *N*-acetyl-l-cysteine (NAC), the precursor of l-cysteine and reduced glutathione as well as a source of sulfhydryl groups in cells. We have shown that treatment with DETC at concentrations that cause a moderate increase in thiol-state imbalance but not cell death stimulated oxidative stress measured as increased level of protein carbonyls, lipid peroxidation, adaptive response of GSH-related enzymes and apoptosis. The increase of cellular GSH level by NAC prevented the observed changes. The results show that cellular effects of DETC are similar to that of disulfiram (Grosicka-Maciąg et al. 2010) and similar mechanisms may explain the effects since the response to both compounds is at least partially attributable to the initial redox cellular state.

## Materials and methods

### Materials

Ham’s F-10 medium (without hypoxanthine and thymidine), newborn calf serum (NCS), penicillin/streptomycin, trypsin–EDTA solution, and phosphate-buffered saline (PBS) were obtained from Gibco BRL. All cell culture plastics were from Becton–Dickinson (San Diego, CA, USA). Diethyldithiocarbamic acid sodium salt trihydrate (DETC) (CAS Register number 20624-25-3) was purchased from MP Biomedicals (Germany) (purity crystalline 99 %). *N*-acetyl-l-cysteine (NAC), dimethyl sulfoxide (DMSO), phenylmethylsulfonyl fluoride (PMSF), 5,5′ -Dithio-bis(2-nitrobenzoic acid) (DTNB), thiobarbituric acid (TBA), 1,1,3,3-tetramethoxypropan (TMP), guanidine HCl, glutathione, sodium dodecyl sulfate (SDS), dinitrophenyl-hydrazine (DNPH), Trypan blue (TB), Thiazolyl Blue Tatrazolium Bromide (MTT), and all other general laboratory chemicals were obtained from Sigma (St Louis, MO, USA).

### Experimental procedures

#### Cell culture conditions and treatments

Chinese hamster V79 lung fibroblasts (clone M8) were purchased from Prof. M.Z. Zdzienicka (Collegium Medicum im. Ludwika Rydygiera, Bydgoszcz, Polska). Cells were grown until 80–90 % confluence under standard conditions in Ham’s F-10 medium (without hypoxanthine and thymidine) in a 95 % air, 5 % CO_2_ humidified incubator at 37 °C. Medium contained 15 % heat-inactivated NCS, penicillin/streptomycin (100 U/ml, 100 μg/ml). Cells were harvested by treatment with 0.25 % trypsin–0.02 % EDTA in PBS and were seeded (2 × 10^6^ cells) in 10 ml complete medium in a 10-cm plate or (3 × 10^6^) in 25 ml complete medium in a 15-cm plate. The cells were then allowed to grow at 37 °C and 5 % CO_2_ for 1–2 days up to 60–70 % confluence prior to treatment. Cells were treated for 1 h with various concentrations of DETC (50, 100, 200, 300 μM) diluted from the stock solution (final concentration of DMSO set to 0.1 %). When pre-treatment of cells was required, 5 mM *N*-acetyl-l-cysteine was applied to V79 cells for 24 h before cells were exposed to DETC. Dosing schedule was analogous to our previous toxicological data from disulfiram (Grosicka-Maciąg et al. 2010). The control group was exposed to an equivalent concentration of solvent. Under these conditions, NAC did not affect cell proliferation or viability. DMSO at this concentration had no effect on cell growth and was used as solvent control for all tested parameters. Specific set of reaction conditions (e.g., control, DETC or NAC, NAC − DETC) was evaluated together on the same day over multiple occasions.

#### Trypan blue exclusion assay

Cytotoxicity was determined by trypan blue (TB) exclusion assay. Cells were treated for 1 h with DETC or pre-incubated with 5 mM NAC by 24 h followed by 1 h treatment with DETC (50, 100, 200, 300 μM). Untreated or NAC-treated cultured cells were used as the 100 % viability value, respectively. The number of all colonies has been determined in three Petri dishes per concentration. Experiments were repeated three times.

#### Cell growth assay

The growth inhibition effect of DETC on cells was determined by measuring MTT day absorbance by living cells as previously described (Grosicka-Maciag et al. 2010).

#### Colorimetric determination of reduced (GSH) and oxidized (GSSG) glutathione

The levels of cellular GSH and GSSG were determined using a standard BIOXYTECH^®^ GSH/GSSG-412™ kit from *Oxis*Research™ according to manufacturer’s protocol, as described previously (Grosicka-Maciąg et al. 2010). Results are expressed as total (reduced and oxidized or GSH_*t*_) and oxidized glutathione (GSSG) as μg/mg protein. Protein concentration was determined by Bradford (1976) protein assay.

#### Protein carbonyl groups (PC) assay

Protein carbonyl groups (PC) were measured using reaction with dinitrophenyl-hydrazine (DNPH), leading to the formation of stable hydrazone products (Reznick and Packer 1994), as described previously (Grosicka et al. 2005). The results are expressed as nmol of carbonyl groups/mg protein.

#### Lipid peroxidation assay

Lipid peroxides were detected as malondialdehyde (MDA) reacting with thiobarbituric acid (TBA) to form a 1:2 adduct. The MDA–TBA colored complex (TBARS) was measured by spectrofluorometric analysis (Miceli et al. 1994), as described previously (Grosicka et al. 2005). The quantities of TBARS were expressed in terms of amount (nmol) per 100 mg protein.

#### Antioxidative enzymes activity determination

Preparation of cell extracts for enzyme assays was obtained as described previously (Grosicka-Maciąg et al. 2010). Cells (7 × 10^7^) were incubated for 1 h with 200 μM DETC without or with NAC pre-treatment.

#### Catalase activity

Catalase (CAT) was determined by the kinetic assay according to method described by Góth (1991). 0.05–0.1 ml sample was incubated with H_2_O_2_ as a substrate at 37 °C for 60 sec. The enzymatic reaction was stopped with ammonium molybdate, and the yellow complex of molybdate and hydrogen peroxide was measured at 405 nm. The measurement of catalase activity is based in the quantification of the hydrogen peroxide breakdown; thus, we define one unit of catalase as the amount of enzyme required to decompose one μmole of H_2_O_2_ per minute at 37 °C. Specific activity of CAT was expressed as U/mg protein.

#### Glutathione peroxidases (GPx) activity

Glutathione peroxidase (GPx) activity was determined according to the method described by Wendel (1981). GPx catalyzes the reduction of various organic hydroperoxides and H_2_O_2_ using reduced glutathione (GSH) as donor:

GPx assay was carried out by monitoring the oxidation of nicotinamide adenine dinucleotide phosphate (NADPH) in a recycling assay as described previously (Grosicka-Maciąg et al. 2010). Activity of total non-Se-dependent GPx and Se-dependent GPx was determined using cumene hydroperoxide (CHP) and H_2_O_2_ as a substrate, respectively. A 50-μl sample was used to measure the GPx activity. Specific activity of GPx was expressed as U/mg protein. One unit of GPx activity was defined as the amount of enzyme that oxidized one μmole NADPH per minute.

#### Glutathione reductase

Glutathione reductase (GR) activity was assayed using oxidized glutathione (GSSG) as substrate (Goldberg and Spooner 1992) as described previously (Grosicka-Maciąg et al. 2010). Specific activity of GR was expressed as U/mg protein. One unit is defined as the amount of enzyme that oxidized one μmole NADPH per minute at 37 °C.

#### Annexin V staining for cell death detection

Apoptosis was determined by TUNEL, a terminal deoxynucleotidyl transferase (TdT)-based end-labeling assay for DNA strand breaks (Li and Darzynkiewicz 1995) and staining cells with dual Annexin V-fluorescein isothiocyanate (FITC) and propidium iodide (PI) binding assays. Cells (0.5 × 10^6^) in 35-mm culture dish were incubated with the indicated amounts of DETC with or without NAC pre-treatment for 1 to 4 h.

#### TUNEL assay

The TUNEL assay was performed using the APO-DIRECT™ test (Becton–Dickinson, San Diego, CA, USA). Briefly, fixed cells were incubated with bromodeoxyuridine triphosphate (Br-dUTP) and TdT, which binds the Br-dUTP to the 3′-hydroxyl end of the RNA fragment. The Br-dUTP was detected using FITC-labeled anti-Br-dUTP monoclonal antibody and the DNA was counterstained with 4,6-diamidino-2-phenylindole dihydrochloride (DAPI) for image analysis. Parallel negative controls with distilled water instead of TdT were run for each sample. To analyze apoptosis-specific DNA fragmentation, cells were treated with 100 or 200 μM DETC (4 h). DETC-mediated nuclear DNA fragmentation was analyzed using Nikon Eclipse E800 fluorescent microscope equipped with Nikon Coolpix 995 digital camera (both from Nikon, Japan). Manufacturer (Becton–Dickinson, San Diego, CA, USA) provided positive controls. Apoptosis induction efficacy was calculated as percentage of fluorescein-positive to DAPI-stained nuclei.

#### Annexin V-binding apoptosis/necrosis discrimination assay

Fluorochrome-labeled Annexin V was used to detect apoptosis. To discriminate between necrotic and apoptotic cells, propidium iodide (PI) was added simultaneously to the cell suspension. The assay was performed according to manufacturer’s protocol (APOPTEST-OregonGreen™ assay, NexinsResearch, Kattendijke, Netherlands) as described previously (Grosicka-Maciąg et al. 2010). Cells after 200 μM DETC treatment (1 h) were washed twice with ice-cold PBS and incubated with Oregon Green™-labeled Annexin V and PI for 30 min. The number of Annexin V- and/or PI-positive cells, corresponding to apoptotic or necrotic events, respectively, was analyzed using Nikon Eclipse E800 fluorescent microscope with Nikon digital camera (Nikon, Japan).

### Statistical analysis

Statistical analysis was performed with a statistical package—Statistica 5.1 software (Statsoft, Warsaw, Poland) using Kolmogarof–Smirnov test to assess data distribution, the Kruskal–Wallis test to compare selected pairs of data. Data are shown as mean ± SD of five to six assays. Statistical significance was considered at *P* < 0.05.

## Results

### Effect of DETC on cell viability using trypan blue (TB) and MTT tests

**Fig. 1:**
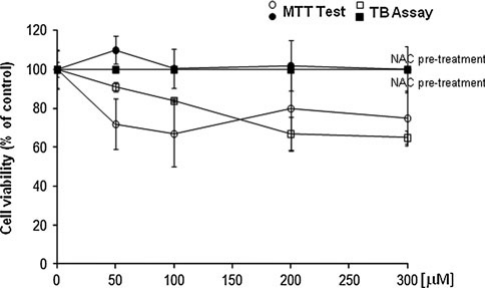
Effect of DETC on cell viability using TB exclusion assay and MTT test. Values are expressed as* percentage* of viable cells with respect to controls. All data represent the mean ± SD of three experiments, each of them performed in triplicate

To determine the cytotoxicity of DETC to V79 cells, cell viability was evaluated based on DETC effect on membrane integrity and on enzymatic activity of mitochondrial dehydrogenases using two tests: trypan blue (TB) exclusion assay and MTT test (Fig. 1). The number of viable cells (TB exclusion assay) decreased in a concentration-dependent manner by 30 % after exposure to 200 μM concentration and was kept at the same level at 300 μM concentration. Decreased amount of purple formazon observed in cells treated with DETC indicates changes in mitochondrial metabolic activity induced by the compound. The decrease by 25 % was seen in MTT test already at 50 μM concentration of DETC, and the decrease was not significantly changed up to 300 μM concentration. Cells pre-incubated with NAC followed by DETC treatment did not show any decrease in the cells viability (TB exclusion assay), as well as any toxicity measured by MTT test, comparing to control V79 cells.

### Effect of DETC on protein oxidation (PC) and on lipid peroxidation level (TBARS)

The increase of protein oxidation, measured as protein carbonyl groups (PC) level, and the increase of lipid peroxidation, measured as TBARS production, were observed only in cells exposed to 200 μM concentration, comparing to control V79 cells (Table 1) (*P* < 0.05 vs. control). At lower, 100 μM concentration, both parameters were not significantly changed comparing to control cells. Since the prooxidative effect of DETC was observed only at 200 μM concentration, we analyzed the effect of increased GSH level after NAC pre-treatment only at this concentration. Pre-incubation of cells with NAC followed by 200 μM DETC exposure prevented DETC-generated protein and lipids oxidative damage.

**Table 1:** Effect of DETC on protein oxidation (PC) and lipid peroxidation (TBARS) without or with NAC pre-treatment

PC (nmol/mg protein)			TBARS (nmol/100 mg protein)		
Control	100 μM	200 μM	Control	100 μM	200 μM
Without NAC pre-treatment					
2.431 ± 0.612	2.316 ± 0.698	3.404 ± 0.689*	57 ± 9	67 ± 25	100 ± 4*
With NAC pre-treatment					
1.947 ± 0.573	NT	2.264 ± 0.225	53 ± 6	NT	56 ± 5

V79 cells were pre-incubated with 5 mM NAC for 24 h and subsequently treated with 100 or 200 μM DETC for 1 h. One group of cells was treated only with: 100, 200 μM DETC for 1 h or with 5 mM NAC for 24 h. Data represent the mean ± SD of six independent samples for each experiment

*NT* not tested

*^^ *P* < 0.05 versus control

### Effect of DETC on intracellular reduced (GSH_*t*_) and oxidized (GSSG) glutathione levels

**Fig. 2:**
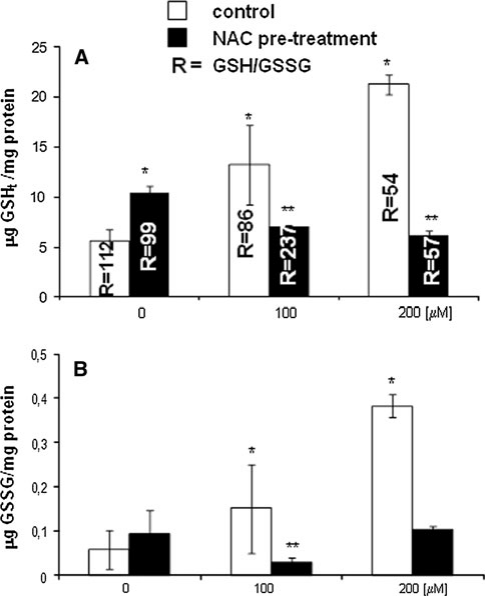
Effect of DETC on GSH and GSSG level without or with NAC pre-treatment. V79 cells were treated only with: (i) 100 μM or 200 μM DETC for 1 h; (ii) with 5 mM NAC for 24 h; (iii) with 5 mM NAC for 24 h followed by 100 μM or 200 μM DETC for 1 h. Data represent the mean ± SD of six independent samples for each experiment. *P < 0.05 versus control cells; **P < 0.05 versus NAC pre-treated cells

As seen in Fig. 2a, b, DETC causes significant, dose-dependent increase in both GSH total (GSH_*t*_) and GSSG levels after 1 h incubation. The level of GSH_*t*_ at 100 and 200 μM concentrations was increased 2.3- and 3.7-fold, respectively, compared to the control value. Similarly, the level of GSSG was increased 2.6- and 6.8-fold, respectively, compared to the control cells. The ratio GSH/GSSG (*R*) was decreased compared to the control value (*R* = 112). R value at 100 and 200 μM DETC was 86 and 54, respectively.

Cells incubated only with NAC for 24 h showed statistically significant increase of GSH_*t*_ and slight increase of GSSG comparing to control cells (Fig. 2a, b); however, *R* value was not significantly changed comparing to the control value (*R* = 99 and *R* = 112, respectively). When the cells were pre-treated with NAC followed by 100 or 200 μM DETC exposure, the protective effect of NAC was observed. GSH_*t*_ and GSSG levels were decreased and restored to the control values. However, *R* value at 200 μM DETC concentration was still decreased and kept at the same level (*R* = 57) as after DETC exposure alone (*R* = 54).

### Effects of DETC on antioxidant enzyme activity

**Fig. 3:**
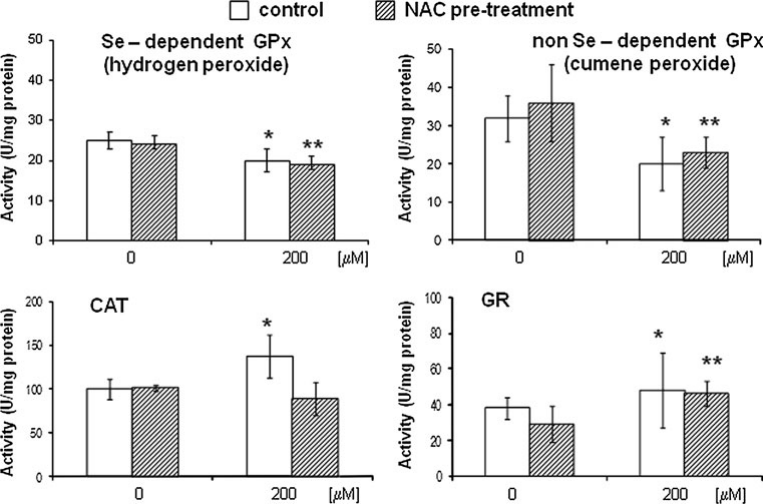
Effect of DETC on antioxidative enzymes activity without or with NAC pre-treatment. V79 cells were pre-incubated with 5 mM NAC for 24 h and subsequently treated with 200 μM DETC for 1 h. One group of cells was treated only with: 200 μM DETC for 1 h or with 5 mM NAC for 24 h. Data represent the mean ± SD of six independent samples for each experiment. *P < 0.05 versus control. **P < 0.05 versus NAC pre-treated cells

In order to understand the mechanism underlying the observed cellular effects of DETC, the activities of CAT and the GSH-related enzymes, such as GPx and GR, were determined in control and in NAC pre-treated cells. The results are presented in Fig. 3. Enzymes activity was measured at 200 μM DETC since the increase in protein oxidation and lipids peroxidation, as well as decrease in GSH/GSSG ratio was most pronounced at that concentration. As shown in Fig. 3, V79 cells exposed to either DETC or DETC after NAC pre-treatment showed significant decreases of the specific activities of both peroxidases: Se-dependent GPx (22 %) (*P* < 0.05) and non-Se-dependent GPx (37 %) (*P* < 0.05). The specific activities measured in cells treated either with DETC or DETC after NAC pre-treatment were compared with the activities measured in control or in NAC treated cells, respectively.

Contrary, the activity of GR and CAT was elevated by DETC exposure and the increase was statistically significant. GR activity in control cells increased by 26 % (*P* < 0.05), comparing to 59 % (*P* < 0.05) increase in cells after NAC pre-treatment, as compared to control or NAC treated cells, respectively. CAT activity was increased by 45 % (*P* < 0.05) by DETC only in cells without NAC pre-treatment.

### DNA fragmentation and Annexin V-binding activation

**Fig. 4:**
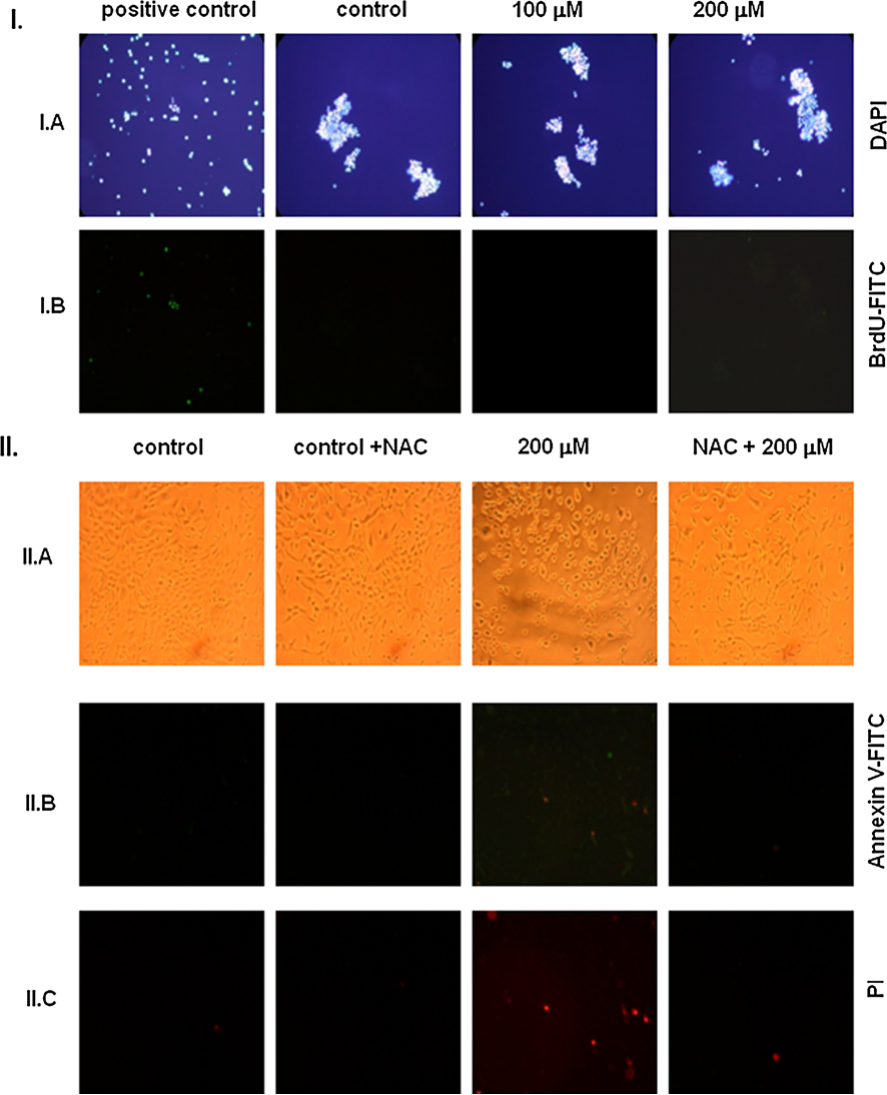
Effect of DETC on apoptosis induction in V79 cells. **I** Control and DETC (100, 200 μM) treated cells were stained with DAPI (**I**.**A**) or BrdU-FITC (**I**.**B**); **II** Control, NAC, DETC (200 μM), and DETC (200 μM) after NAC pre-treatment cells seen in phase—contrast microscope (**II**.**A**), the same cells after staining with Annexin V (**II**.**B**) or IP (**II**.**C**). Representative areas were photographed under Nikon Eclipse E800 fluorescent microscope equipped with Nikon Coolpix 995 digital camera using ×500 magnifications. Time treatment with DETC was 4 h for TUNEL assay (**I**.**A**; **I**.**B**), and for 1 h for Annexin V/PI assay (**II**.**B**, **II** **C**)

To determine whether oxidative changes induced by DETC are concomitant with apoptosis in V79 cells, we investigated DNA fragmentation, which is one of biochemical hallmarks of apoptosis. Analysis of DNA fragmentation, as assessed by the TUNEL assay with DAPI (Fig. 4IA) or BrdU/FITC (Fig. 4IB), revealed that exposure to DETC resulted in the same level of fluorescein-stained nuclei (corresponding to extensive DNA fragmentation) as compared to untreated control.

The analysis of phosphatidylserine (PS) externalization, using a dual staining of cells with Annexin V-Oregon Green (Fig. 4IIB) and propidium iodine (PI) (Fig. 4IIC), revealed 90 % of apoptotic cells with green fluorescence after exposure to DETC and only a few cells (10 from 300) with red fluorescence characteristic for late apoptosis/necrosis. NAC pre-treatment protected cells against pro-apoptotic activity of DETC. A number of observed apoptotic and necrotic cells were at the level of control untreated cells.

## Discussion

*N,N*-Diethyldithiocarbamate besides being a main metabolite of disulfiram, the drug used over half century for alcohol aversion therapy, has diverse applications, also as pharmacological agent. One molecule of disulfiram gives two molecules of DETC by the reduction of intramolecular disulfide bond. Recently, we have shown that oxidative properties of disulfiram are at least partially attributable to its cellular effect (since mechanisms induced by NAC pre-treatment lower or even abolish the observed effect of DSF on GSSG level, protein carbonyl content, and its pro-apoptotic activity) (Grosicka-Maciąg et al. 2010). In the present study, we took into account the role of oxidative stress in mechanism of DETC cytotoxicity. The results of the present work show that effects of DETC and disulfiram may be explained by the same cellular mechanisms since they are at least partially attributable to the initial redox cellular state.

We have shown that DETC cytotoxicity depends on the initial conditions in term of intracellular glutathione level. Cell viability was restored to the control values after NAC pre-treatment followed by DETC exposure. Our study showed decrease of GSH/GSSG ratio (*R*) in cells treated with DETC comparing with the control cells. Decrease of GSH/GSSG ratio was revealed despite an increase of GSH level observed in cells treated with DETC. Fact of concomitant GSSG generation by DETC, potentially harmful for cells, is consistent with mutual interconversion with disulfiram (Burkitt et al. 1998). Deneke et al. (1997) observed that increase in intracellular GSH after addition of DETC to the culture media was associated with an increase in the rate of cysteine uptake into the cells. Observed GSH/GSSG depletion affected oxidative damage of proteins and lipids in V79 cells. Other groups revealed also an increase in PC level in proteins isolated from DETC-exposed rats (Viquez et al. 2007) as well as membrane lipid peroxidation (Lushchak et al. 2007; Tonkin et al. 2004; Valentine et al. 2009; Viquez et al. 2008). Contrary to prooxidative properties of DETC, there are studies revealing antioxidative properties of the drug (Liu et al. 1996). Koster and van Berkel (1983) and Schreck et al. (1992) revealed that DETC inhibits lipid peroxidation and hydroxyl radicals in rats. Mülsch et al. (1993) showed inhibition of nitric oxide synthase induction in macrophage by DETC.

Fact, that observed protein oxidation and lipid peroxidation by DETC has been prevented by pre-incubation of cells with NAC, suggests that reduced cell environment is crucial for revealing DETC oxidative properties. The protective effect of GSH may result from preventing cellular reduced sulfhydryls from undergoing mixed disulfide reaction with DSF produced in mutual reaction with DETC.

Since maintaining of optimal GSH/GSSG ratio in the cell is critical to survival, a tight regulation of the system is essential. GSH content within a cell can be increased due to adaptive mechanisms to oxidative stress through an increase in GSH synthesis or enzymatic reduction of its oxidized form. However, a severe oxidative stress may suppress GSH level due to the loss of adaptive mechanisms and the oxidation of GSH to GSSG (Townsend et al. 2003). In the present studies, we investigated the antioxidant enzyme activities. Analysis of GSH-related enzymes showed a significant increase of glutathione reductase (GR) activity and a decrease of both glutathione peroxidases (GPx) activity. These enzymes are partially responsible for determining the susceptibility of cells to oxidative stress (Yang et al. 2006). It is believed that activity of GR may be the major determinant that regulates GSH/GSSG ratio (Argyrou and Blanchard 2004). Rogers et al. (2004) observed that exposure to agents that lead to increased oxidative stress also leads to an increase in GR mRNA content. Observed increase of GR activity in cells exposed to DETC could be a reason of GSH augmentation. It is interesting that the highest increase of GR activity was observed in cells after NAC pre-treatment as compared to corresponding control. Simultaneously, we observed a decrease of both total non-Se-dependent and Se-dependent GPx after DETC exposure. NAC pre-treatment had no effect on both forms GPx in DETC-exposed cells. Blum and Fridovich (1985) described that GPx activity may be inactivated in oxidative stress conditions, and toxic ligands, such as MDA, can partially inhibit GPx activity (Arshad et al. 1991). With the lowered GPx activities in DETC-treated cells, the enzyme does probably not detoxify H_2_O_2_ or other hydroperoxides completely. Catalase is another enzyme, besides GPx, that converts H_2_O_2_ to H_2_O. The increase of CAT activity observed in cells exposed to DETC could be explained by adaptive response of the cell to elevated level of H_2_O_2_. Lushchak et al. (2007), Prabhu and Nandini (2007) have shown similar effects of DETC exposure on ROS scavenging system. H_2_O_2_ concentration is then controlled within a cell by coordinated action of two enzymes: Se-GPx and CAT.

We investigated the hallmarks of apoptosis in V79 cells, since oxidative changes induced by many drugs may be concomitant with the cell death. Analysis of DNA fragmentation in V79 cells revealed that DETC does not induce DNA fragmentation in V79 cells; however, the analysis of another hallmark of apoptosis, phosphatidylserine (PS) externalization has shown an increased number of cells with externalized PS. The number of apoptotic and necrotic cells was significantly decreased in V79 cells after NAC pre-treatment followed by DETC exposure. In our present studies, the DETC effects on apoptosis are similar to our earlier observation with disulfiram-induced cytotoxicity and apoptosis (Grosicka-Maciąg et al. 2010). The results show a correlation of induced apoptosis with the above-mentioned changes in both forms GSH and GSSG. Since pre-incubation with NAC significantly reversed the apoptotic effect of DETC in V79 fibroblasts, we conclude that the level of glutathione or/and another thiol redox buffer is likely to be involved in the apoptosis/necrosis processes induced by DETC. Among several studies on the regulation of apoptosis by DETC, it has been shown that the drug can reveal pro- or antiapoptotic activities depending on type of cells used. Our results are in agreement with other papers (Han et al. 2008; Han and Park 2009; Kang et al. 2001). However, Dumay et al. (2006) showed antiapoptotic activity of DETC. On the other hand, Kimoto-Kinoshita et al. (2004) observed that DETC induces two different types of death: apoptosis and necrosis in human promyelocytic leukemia (HL60) cells depending on the drug concentrations used. Various effects of DETC on cell death are mediated by intracellular redox regulation and/or the different MAP kinase activation. Similarly, Kanno et al. (2003) have found induction of different types of cytotoxicity and apoptosis by DETC in various human and murine leukemia cell lines. The authors suggest that the DETC-induced cytotoxicity and DNA fragmentation were triggered by the depletion of intracellular GSH and accompanied by the activation of endonuclease. The changes in intracellular GSH level are also the reason of apoptosis induction in HeLa cells after DETC treatment (Han et al. 2008). Similarly, Han and Park (2009) showed increased GSH depletion and apoptosis in Calu-6 lung cells after exposure to DETC.

In conclusion, the initial GSH level is essential in the modulation of cytotoxic effects observed in cells exposed to DETC. Intensity of cellular effects observed in cells exposed to DETC was comparable to those observed in cells exposed to disulfiram and may be explained by similar mechanisms, since the response to both compounds is at least partially attributable to the initial redox cellular state. However, DETC and DSF differ in the intensity of induced cellular effects. It seems disulfiram as disulfide of DETC is a much safer drug than DETC. However, it is very difficult to answer the question whether a potential clinical use of DETC would provide advantages or rather be disadvantageous.

Additionally, we would like to indicate that V79 cells used as a model system for cytotoxicity studies are quite distant from cells in the organism, because they are deficient of cytochromes P450 that are also involved in the generation of reactive oxygen species. Therefore, we recommend that the results obtained in the present study should be verified in primary cells such as primary hepatocytes.

## References

[CR1] Argyrou A, Blanchard JS (2004). Flavoprotein disulfide reductases: advances in chemistry and function. Prog Nucleic Acid Res Mol Biol.

[CR2] Arnelle DR, Day BJ, Stamler JS (1997). Diethyl dithiocarbamate-induced decomposition of S-nitriosothiols. Nitric Oxide.

[CR3] Arshad MA, Bhadra S, Cohen RM, Subbiah MT (1991). Plasma lipoprotein peroxidation potential: a test to evaluate individual susceptibility to peroxidation. Clin Chem.

[CR4] Arthur JR (2000). The glutathione peroxidases. Cell Mol Life Sci.

[CR5] Blum J, Fridovich I (1985). Inactivation of glutathione peroxidase by superoxide radical. Arch Biochem Biophys.

[CR6] Bradford M (1976). A rapid and sensitive method for the quantitation of microgram quantities of protein utilizing the principle of protein dye binding. Anal Biochem.

[CR7] Bradley M, Bhuyan B, Francis MC, Langenbach R, Peterson A, Huberman E (1981). Mutagenesis by chemical agents in V79 Chinese hamster cells: a review and analysis of literature. Mutat Res.

[CR8] Burkitt MJ, Bishop HS, Milne L, Tsang SY, Provan GJ, Nobel CS, Orrenius S, Slater AF (1998). Dithiocarbamate toxicity toward thymocytes involves their copper-catalyzed conversion to thiuram disulfides, which oxidize glutathione in a redox cycle without the release of reactive oxygen species. Arch Biochem Biophys.

[CR9] Byrne ST, Gu P, Zhou J, Denkin SM, Chong C, Sullivan D, Liu JO, Zhang Y (2007). Pyrrolidine dithiocarbamate and diethyldithiocarbamate are active against growing and nongrowing persister *Mycobacterium tuberculosis*. Antimicrob Agents Chemother.

[CR10] Deneke SM, Harford PH, Lee KY, Deneke CF, Wright SE, Jenkinson SG (1997). Induction of cysteine transport and other stress proteins by disulfiram: effects on glutathione levels in cultured cells. Am J Respir Cell Mol Biol.

[CR11] Dumay A, Rincheval V, Trotot P, Mignotte B, Vayssiere J-L (2006). The superoxide dismutase inhibitor diethyldithiocarbamate has antagonistic effects on apoptosis by triggering both cytochrome c release and caspase inhibition. Free Radic Biol Chem.

[CR12] Fitsanakis VA, Amarnath V, Moore JT, Montine KS, Zhang J, Montine TJ (2002). Catalysis of catechol oxidation by metal-dithiocarbamate complexes in pesticides. Free Radic Biol Med.

[CR13] Goldberg DM, Spooner RJ, Bergmayer HU (1992). Glutathione reductase. Methods of enzymatic analysis.

[CR14] Góth L (1991). A simple method for determination of serum catalase activity and revision of reference range. Clin Chim Acta.

[CR15] Grosicka E, Sadurska B, Szumiło M, Grzela T, Łazarczyk P, Niderla-Bielińska J, Rahden-Staroń I (2005). Effect of glutathione depletion on apoptosis induced by thiram in Chinese hamster fibroblasts. Int Immunopharmacol.

[CR16] Grosicka-Maciąg E, Kurpios-Piec D, Grzela T, Czeczot H, Skrzycki M, Szumiło M, Rahden-Staroń I (2010). Protective effect of N-acetylcysteine against disulfiram induced oxidative stress and apoptosis in V79 cells. Toxicol Appl Pharmacol.

[CR17] Han YH, Park WH (2009). The effects of N-acetyl cysteine, buthionine sulfoximine, diethyldithiocarbamate or 3-amino-1,2,4-triazole on antimycin A-treated Calu-6 lung cells in relation to cell growth, reactive oxygen species and glutathione. Oncol Rep.

[CR18] Han YH, Kim SZ, Kim SH, Park WH (2008). Enhancement of arsenic trioxide-induced apoptosis in HeLa cells by diethyldithiocarbamate or buthionine sulfoximine. Int J Oncol.

[CR19] Hosni M, Meskini N, Prigent AF, Anker G, Joulain C, el Habib R, Lagarde M (1992). Diethyldithiocarbamate (ditiocarb sodium) effect on arachidonic acid metabolism in human mononuclear cells. Glutathione peroxidase-like activity. Biochem Pharmacol.

[CR20] Johansson B (1992). A review of the pharmacokinetics and pharmacodynamics of disulfiram and its metabolites. Acta Psychiatr Scand.

[CR21] Kang JH, Wei YM, Zheng RL (2001). Effects of diethyldithiocarbamate on proliferation, redifferention, and apoptosis in human hepatoma cells. Acta Pharmacol Sin.

[CR22] Kanno S, Matsukawa E, Miura A, Shouji A, Asou K, Ishikawa M (2003). Diethyldithiocarbamate-induced cytotoxicity and apoptosis in leukemia cell lines. Biol Pharm Bull.

[CR23] Kimoto-Kinoshita S, Nishida S, Tomura TT (2004). Diethyldithiocarbamate can induce two different type of death: apoptosis and necrosis mediating the differential MAP kinase activation and redox regulation in HL60 cells. Mol Cell Biochem.

[CR24] Kishore K, Moorthy PN (1991). Nature of the transient species in the one-electron oxidation of diethyldithiocarbamate as studied by pulse radiolysis. J Chem Soc Perkin Trans.

[CR25] Koster JF, van Berkel TJ (1983). The effect of diethyldithiocarbamate on the lipid peroxidation of rat-liver microsomes and intact hepatocytes. Biochem Pharmacol.

[CR26] Li X, Darzynkiewicz Z (1995). Labeling DNA strand breaks with BrdUTP. Detection of apoptosis and cell proliferation. Cell Prolif.

[CR27] Liu J, Shigenaga MK, Yan LJ, Mori A, Ames BN (1996). Antioxidant activity of diethyldithiocarbamate. Free Radic Res.

[CR28] Lushchak V, Semchyshyn H, Lushchak O, Mandryk S (2005). Diethyldithiocarbamate inhibits in vivo Cu, Zn-superoxide dismutase and perturbs free radical processes in the yeast Saccharomyces cerevisiae cells. Biochem Biophys Res Commun.

[CR29] Lushchak V, Bagnyukova TV, Lushchak O, Storey JM, Storey KB (2007). Diethyldithiocarbamate injection induces transient oxidative stress in goldfish tissues. Chem Biol Interact.

[CR30] Mankhetkorn S, Abedinzadeh Z, Houee-Levin C (1994). Antioxidant action of sodium diethyldithiocarbamate: reaction with hydrogen peroxide and superoxide radical. Free Radical Biol Med.

[CR31] Miceli MV, Liles MR, Newsome A (1994). Evaluation of oxidative processes in human pigment epithelial cells associated with retinal outer segment phagocytosis. Exp Cell Res.

[CR32] Mülsch A, Schray-Utz B, Mordvintcev PI, Hauschildt S, Busse R (1993). Diethyldithiocarbamate inhibits induction of macrophage NO synthase. FEBS Lett.

[CR33] Orrenius S, Nobel CS, van den Dobbelsteen DJ, Burkitt MJ, Slater AF (1996). Dithiocarbamates and the redox regulation of cell death. Biochem Soc Trans.

[CR34] Pande V, Ramos MJ (2003). Nuclear factor kappa B: a potential target for anti-HIV chemotherapy. Curr Med Chem.

[CR35] Pang H, Chen D, Cui QC, Dou QP (2007). Sodium diethyldithiocarbamate, an AIDS progression inhibitor and a copper-binding compound, has proteasome-inhibitory and apoptosis-inducing activities in cancer cells. Int J Mol Med.

[CR36] Prabhu HR, Nandini M (2007). Inhibition of selenium dependent glutathione peroxidase and superoxide dismutase in rats by diethyldithiocarbamate: effect of pre-administration of alfa-tocopherol. Indian J Exp Biol.

[CR37] Reznick AZ, Packer L (1994). Oxidative damage to proteins: spectrophotometric method for carbonyl assay. Methods Enzymol.

[CR38] Rogers LK, Tamura T, Rogers BJ, Welty SE, Hansen TN, Smith CV (2004). Analyses of glutathione reductase hypomorphic mice indicate a genetic knockout. Toxicol Sci.

[CR39] Schreck R, Meier B, Männel DN, Dröge W, Baeuerle PA (1992). Dithiocarbamates as potent inhibitors of nuclear factor kappa B activation in intact cells. J Exp Med.

[CR40] Somers PK, Medford RM, Saxena U (2000). Dithiocarbamates: effects on lipid hydroperoxides and vascular inflammatory gene expression. Free Radic Biol Med.

[CR41] Tonkin EG, Valentine HL, Milatovic DM, Valentine WM (2004). N, N,-diethyldithiocarbamate produces copper accumulation, lipid peroxidation, and myelin injury in rat peripheral nerve. Toxicol Sci.

[CR42] Townsend DM, Tew KD, Tapiero H (2003). The importance of glutathione in human disease. Biomed Pharmacother.

[CR43] Valentine HL, Viquez OM, Amarnath K, Amarnath V, Zyskowski J, Kassa EN, Valentine WM (2009). Nitrogen substituent polarity influences dithiocarbamate-mediated lipid oxidation, nerve copper accumulation, and myelin injury. Chem Res Toxicol.

[CR44] Viquez OM, Valentine HL, Friedman DB, Olson SJ, Valentine WM (2007). Peripheral nerve protein expression and carbonyl content in N, N-diethyldithiocarbamate myelinopathy. Chem Res Toxicol.

[CR45] Viquez OM, Valentine HL, Amarnath K, Milatovic D, Valentine WM (2008). Copper accumulation and lipid oxidation precede inflammation and myelin lesions in N, N-diethyldithiocarbamate peripheral myelinopathy. Toxicol Appl Pharmacol.

[CR46] Wendel A (1981). Glutathione peroxidase. Methods Enzymol.

[CR47] Yang MS, Chan HW, Yu LC (2006). Glutathione peroxidase and glutathione reductase activities are partially responsible for determining the susceptibility of cells to oxidative stress. Toxicology.

[CR48] Zanocco AL, Pavez LA, Videla LA, Lissi EA (1989). Antioxidant capacity of diethyldithiocarbamate in a metal independent lipid peroxide process. Free Radic Biol Med.

